# Process performance determination data in thiocyanate biodegradation systems: Use of sulphate production

**DOI:** 10.1016/j.dib.2018.01.017

**Published:** 2018-01-20

**Authors:** Lukhanyo Mekuto, Lovasoa C. Razanamahandry, Seteno K.O. Ntwampe, John-Baptist N. Mudumbi, Gift Muchatibaya

**Affiliations:** a Bioresource Engineering Research Group (BioERG), Department of Biotechnology, Cape Peninsula University of Technology, PO Box 652, Cape Town 8000, South Africa; b International Institute for Water and Environmental Engineering (2iE), Department of Water and Sanitation, Laboratory of Water, Decontamination, Ecosystem and Health (LEDES), 01 P.O. Box 594, 01 Ouagadougou, Burkina Faso; c Department of Mathematics, University of Zimbabwe, Harare, Zimbabwe

**Keywords:** Biodegradation, Sulphates, Thiocyanate, Fluorescein diacetate, Heterotrophic cell counts, Direct cell counts

## Abstract

This data article presents the utilization of sulphates as an indirect technique for the assessment of microbial growth, activity and SCN^-^ biodegradation efficiency since the TDO were observed to be unable to utilise the produced sulphates as a source of sulphur (Mekuto e al., 2017) [1] The TDO demonstrated complete SCN^-^ biodegradation while also utilizing the produced ammonium. The production of SO_4_^2-^ from SCN^-^ biodegradation had a good correlation in comparison to the traditional methods of assessing microbial growth and activity i.e. direct cell counts (DCC), heterotrophic counts (CFU) and fluorescein production from fluorescein diacetate (FDA). The concentration of the produced SO_4_^2-^ demonstrated a similar logarithmic trend with the FDA, DCC and CFU techniques, thus confirming that the production of SO_4_^2-^ from SCN^-^ biodegradation systems can be utilised as an indirect technique for the assessment of microbial growth, activity and SCN^-^ biodegradation performance.

**Specifications Table**

**Table:** 

Subject area	*Environmental Biotechnology*
More specific subject area	*Bioremediation*
Type of data	*Table and Figures*
How data was acquired	*Spectrophotometric determinations were conducted in a Jenway 6715 UV/Vis spectrophotometer while for the Thoma counting chamber (Hawksley, UK) was used for direct cell counts.*
Data format	*Analyzed*
Experimental factors	*Sulphate production from biodegradation of wastewater containing thiocyanate in comparison to traditional microbial growth/activity techniques i.e. Fluorescein diacetate (FDA), Heterotrophic cell counts (CFU), Direct cell counts (DCC).*
Experimental features	*Thiocyanate degrading organisms (TDO) were isolated and identified as described in**[2]**. TDO's were grown for 98 h in SCN*^*-*^*containing solution where the*${\mathrm{SO}}_{4}^{2-}$*production was compared to traditional microbial growth/activity techniques such that*${\mathrm{SO}}_{4}^{2-}$*can be utilized as an indirect assessment of thiocyanate biodegradation system performance. The experiments were conducted in triplicates.*
Data source location	*Cape Town, South Africa (33.9324°S, 18.6406°E)*
Data accessibility	*Data is available in the article*

**Value of the data**

• This research data provides a rapid microbial performance assessment technique in thiocyanate biodegradation systems.
• The data can be utilized by researchers who are active in the development of a robust biological method for the bioremediation of thiocyanate that originates from mining, gasification and coking wastewaters.
• The presented data had a good correlation with the tested traditional techniques that are commonly utilized in microbiology and hence it can be utilized as an indicator for thiocyanate biodegradation process performance.

## 1 Data

**Fig. 1:**
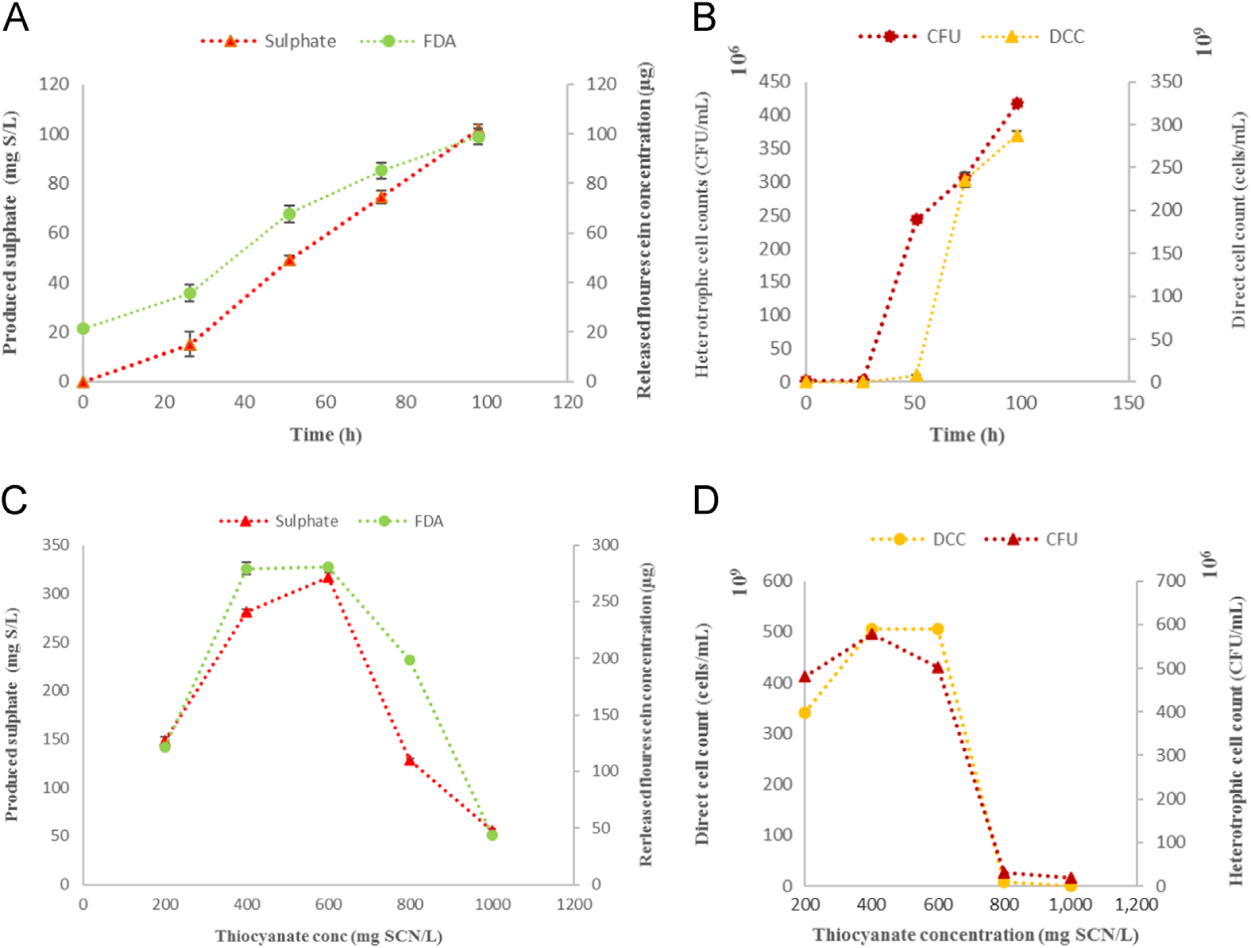
Graphical profiles of sulphate production in comparison to the (a) FDA method, (b) heterotrophic and direct cell counts.

The data presented here demonstrates the utilization of sulphates as an indirect determination of thiocyanate biodegradation systems’ performance. Table 1 demonstrates the biodegradation of SCN^-^ by the TDO with the subsequent production of ammonium while Fig. 1 illustrates the production of sulphates in comparison to the tested traditional microbial growth/activity techniques.

**Table 1:** Thiocyanate degradation and ammonium formation during experimentation.

Measured parameters	Time (h)				
	0	26.5	51.2	74.0	98.0
Biodegraded SCN^-^ (mg/L)	160.42	156.25	33.47	1.67	0.07
${\mathrm{Produced}\;\mathrm{NH}}_{4}^{+}-N\;\left(\text{mg}/L\right)$	0.00	9.25	68.35	32.50	24.90

## 2 Experimental design, materials and methods

### 2.1 Microbial culture and growth conditions

Thiocyanate degrading organisms (TDO) were isolated from SCN^-^ containing wastewater. The TDOs were identified using the 16S rRNA amplicon gene sequencing approach to reveal the bacterial species that were present within the organisms [1]. The procedure and data that was utilised to identify the TDOs were published in [2]. The culture was grown in minimal media as described in [3]. The media was supplemented with SCN^-^, to achieve a concentration of 150 mg SCN^-^/L. The flasks were incubated in an orbital shaker at 30 °C and 180 rpm. Samples were collected at various time intervals and centrifuged at 10,000*g* for 5 min prior to the analysis of SCN^-^ and ${\mathrm{SO}}_{4}^{2-}$. SCN^-^ biodegradation and ${\mathrm{SO}}_{4}^{2-}$ production was evaluated using the ferric iron method [4] and turbidimetrically using barium salts [5] respectively. NH_4_^+^ nitrogen was determined according to the method developed elsewhere [6]. The effect of the initial SCN^-^ concentration on ${\mathrm{SO}}_{4}^{2-}$ production, microbial growth and activity was also assessed in batch cultures for a period of 98 h.

### 2.2 Assessing microbial activity using fluorescein diacetate (FDA)

The microbial activity of the microbial cultures within the biodegradation processes was assessed using the FDA method described in [7]. Briefly, this method works on the hydrolysis of FDA intracellularly by active microorganisms, releasing a fluorescent fluorescein which can be detected spectrophotometrically [8].

### 2.3 Heterotrophic plate counting

The number of viable cells were estimated using the heterotrophic plate count procedure. The plate count procedure was achieved with autoclaved nutrient agar (Meat extract 1.0 g/L, peptone 5.0 g/L, yeast extract 2.0 g/L, NaCl 8.0 g/L, agar 15 g/L). Samples were collected particular intervals and an aliquot (200 µL) of the culture was spread plated after appropriate dilutions. This was followed by incubation at 30 °C for 48 h. The visible microbial colonies were counted as colony forming units (CFU).

### 2.4 Direct cell counting (DCC)

DCC was achieved by the utilisation of a Thoma counting chamber (Hawksley, UK). 5 µl of the planktonic microorganisms sampled from the SCN^-^ degradation experiments were evenly distributed in the chamber and counted directly in an Olympus CX21 phase contrast light microscope (New York, U.S.A.). The mobile cells were the only cells that were counted as these were considered to be active and/or viable while the non-mobile cells were ignored as they were considered to be non-viable or dead.
