# Supplementary material for: Process performance determination data in thiocyanate biodegradation systems: Use of sulphate production
Source: Data Brief. 2018 Jan 20;17:275–8. doi: 10.1016/j.dib.2018.01.017 (PMC5988291; doi:10.1016/j.dib.2018.01.017)

Conflict of interest statement

Process performance determination data in thiocyanate biodegradation systems: Use of sulphate production.

Dear Editor

The authors of the aforementioned manuscript declare that there is no conflict of interest that is associated with the submitted manuscript. That the manuscript has not been submitted to other journals for possible publications while it is submitted in *Data in Brief*.

Lukhanyo Mekuto

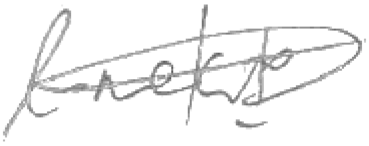

Seteno K.O. Ntwampe

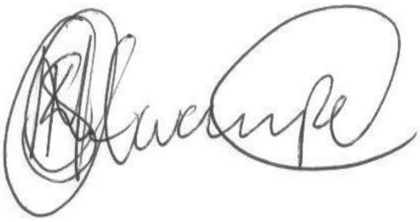

Lovasoa C. Razanamahandry

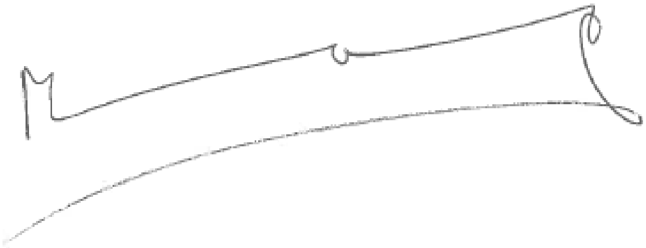

John-Baptist N. Mudumbi

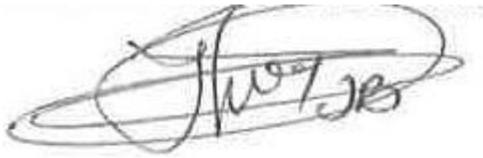

Gift Muchatibaya

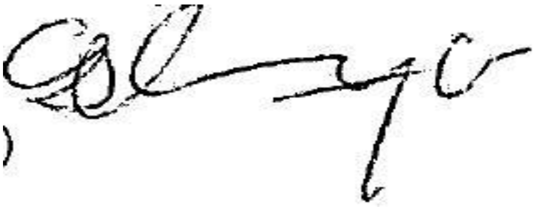

Supplement: Supplementary file 1 — Supplementary material [file mmc1.pdf]
